# Mesenchymal stem cells proliferation and remote manipulation upon exposure to magnetic semiconductor nanoparticles

**DOI:** 10.1016/j.btre.2020.e00435

**Published:** 2020-02-11

**Authors:** Tudor Braniste, Vitalie Cobzac, Polina Ababii, Irina Plesco, Simion Raevschi, Alexandru Didencu, Mihail Maniuc, Viorel Nacu, Ion Ababii, Ion Tiginyanu

**Affiliations:** a National Center for Materials Study and Testing. Technical University of Moldova, Stefan cel Mare av. 168, Chisinau, 2004, Republic of Moldova; b Laboratory of Tissue Engineering and Cells Cultures. State University of Medicine and Pharmacy “Nicolae Testemiteanu”, Stefan cel Mare av. 165, Chisinau, 2004, Republic of Moldova; c Department of Otorhinolaryngology. State University of Medicine and Pharmacy “Nicolae Testemiteanu”, Stefan cel Mare av. 165, Chisinau, 2004, Republic of Moldova; d Department of Physics and Engineering, State University of Moldova, Alexei Mateevici str. 60, Chisinau, 2009, Republic of Moldova; e Academy of Sciences of Moldova, Stefan cel Mare av. 1, Chisinau, 2001, Republic of Moldova

**Keywords:** Mesenchymal stem cells, Nanoparticles, Gallium nitride, Cells guiding

## Abstract

In this paper, we report on spatial redistribution of bone marrow mesenchymal stem cells loaded with magnetic nanoparticles under the influence of continuously applied magnetic field. Semiconductor nanoparticles were synthesized by epitaxial growth of a GaN thin layer on magnetic sacrificial core consisting of ZnFe_2_O_4_ nanoparticles. Different quantities of nanoparticles were incubated *in vitro* with mesenchymal stem cells. High density of nanoparticles (50 μg/ml) leads to a decrease in the number of cells during incubation, while the density of nanoparticles as low as 10 μg/ml is enough to drag cells in culture and rearrange them according to the spatial distribution of the magnetic field intensity.

## Highlights

• The synthesis of GaN/Fe nanoparticles using HVPE method.
• Mesenchymal stem cells interact with GaN-based nanoparticles.
• GaN/Fe nanoparticles rearrange cells under the influence of magnetic field.

## 1 Introduction

Stem cells are defined by their ability to self-renewal and differentiation into more specialized cells, depending on their level of potency [1]. Being easily harvested and cultured, Mesenchymal Stem Cells (MSC) are among the most used stem cells in regenerative medicine. MSC can differentiate into bone, cartilage, fat, tendon, and other different cell lines depending on the given conditions and growth factors [2]. MSC therapeutic potential is enormous and covers a wide medical area, from bone marrow transplantation in blood cancers [3] to the treatment of various diseases like osteoarthritis [4], Crohn`s disease [5], diabetes [6], cardiovascular diseases [7], otorhinolaryngologic pathologies [8], etc. There are several major issues in the therapeutic processes including the non-invasive cellular imaging in high quality transport of therapeutic agent, as well as the remote manipulation in the desired area. The success rate of the therapy based on stem cells depends on the accuracy the cells are delivered to the site of interest. Magnetic resonance imaging (MRI) represents an important tool in cells tracking [9]. The MRI usually includes gadolinium-based contrast agents. Over the last years, however, different types of paramagnetic nanoparticles have been used for tracking cells [10]. The advantage of using nanoparticles for cells tracking relies on the possibility of tuning with the material concentration and chemical composition. Besides tracking, nanoparticles offer the possibility to manipulate with cells and control their spatial position *in vivo* [11]. The superparamagnetic iron oxide nanoparticles (SPION), usually stabilized by coating with dextran, gelatine, chitosan or other polymers, are among the most commonly used nanoparticles for cells tracking [12,13]. Our previous investigations have shown that uncoated gallium nitride nanoparticles (GaN) do not affect the viability and proliferation of endothelial cells [14] and can be used for multifunctional therapeutic purposes which include cells tracking [15]. The use of piezoelectric nanoparticles in combination with magnetic ones could increase the impact of smart materials not only in remote cells imaging but also in the control of the cellular metabolic activity [[16], [17], [18], [19]].

In this work, we report on the interaction of rat MSC with magnetic nanoparticles based on iron covered with a chemically stable crystalline GaN film. The metabolic activity of cells after exposure to nanoparticles was assessed using the MTT assay, which shows no cytotoxic effect during three days of incubation. The spatial redistribution of cells loaded with nanoparticles under a continuous magnetic field was also achieved.

## 2 Materials and methods

### 2.1 Nanoparticles synthesis and characterization

Nanometre-scale thin layers of GaN have been grown on sacrificial zinc ferrite (ZnFe_2_O_4_) based nanoparticles acquired from Sigma-Aldrich (CAS#12063-19-3). The growth took place in a horizontal hydride vapor phase epitaxy (HVPE) reactor with four temperature zones. In the source zone, at T = 850 °C, the GaCl is formed after the interaction of HCl with metallic Ga. Then, in the reaction zone at 600 °C, the formed GaCl interacts with NH_3_ for 10 min in order to initiate the GaN growth on the ZnFe_2_O_4_ nanoparticles. The GaN layer growth accompanied by simultaneous reduction of the ZnO sacrificial substrate nanoparticles occurs at 800 °C for 10 min in the H_2_ flow rate of 3.6 l/min. During the growth process, the ammonia and hydrogen chloride flow were kept constant at 500 ml/min and 15 ml/min, respectively. The initial nanoparticles, as well as the resulted material after the GaN growth, have been characterized using the electron microscopy tools, including scanning electron microscopy (SEM) and transmission electron microscopy (TEM).

### 2.2 Mesenchymal stem cells isolation and culture

The isolation, culture, and use of rat MSC in the research activities were approved by the Ethics Committee of the Moldovan State University of Medicine and Pharmacy “Nicolae Testemitanu” on 18.06.2015.

The MSC were isolated from bone marrow of 5 months old Wistar male rat. After rat euthanasia, the bone marrow from long tubular bones has been flushed with warm PBS (HiMedia, India). The suspension was centrifuged for 10 min at 170 *g*, followed by MSC isolation in mesenchymal stem cells expansion medium HiMesoXL (HiMedia, India) supplemented with antibiotics and antimycotics. The incubation was performed in 25 cm^2^ cell culture flasks (Nunc, Denmark) at 37 °C with 5% CO_2_. The cells were cultured in 2 passages followed by cryopreservation by 5 × 10^5^ cells/ml in FBS (Lonza, Belgium) with 10 % DMSO (OriGen Biomedical, Germany). The MSC isolation and identification was done following the chondrocytes line differentiation protocol [20].

To perform the experiment, 5 × 10^5^ MSC were cultured in 75 cm^2^ culture flasks (Nunc, Denmark) with 15 ml DMEM/Ham's F-12 medium (Sigma, UK) supplemented with 10 % FBS (Lonza, Belgium) and antibiotic antimycotic solution. The medium was completely changed every 2 days until the culture gained confluence. After trypsinization, the cells were counted in hemocytometer with Trypan blue exclusion, followed by cells seeding at a density of 1 × 10^4^ cells/ml in 24 well tissue culture test plates (TPP, Switzerland) for MTT cell viability assay.

### 2.3 Cells exposure to nanoparticles

Different quantities (10, 25, 50 μg/ml) of sterile nanoparticles were homogeneously distributed into the culture media during one hour in an ultrasound bath (Bandelin Sonorex 35 KHz, 120 W). After sonication, the culture media supplemented with nanoparticles were mixed with MSC (10^4^ cells/ml), plated in standard 24 well plates (TPP, Switzerland), and incubated for the next three days at 37 °C, 5% CO_2_. After the monolayer of cells reaches the confluence, the cells loaded with nanoparticles are detached from the culture plate and reseeded under the magnetic field influence.

### 2.4 MTT assay

The MTT assay started 24 h after the medium supplemented with nanoparticles was added to 24 well tissue culture test plates seeded with MSC and has been performed every day during the incubation period (n = 3). The culture medium was replaced by 1 ml of 2.5 mg/ml MTT (Sigma, UK) solution prepared in DMEM/Ham`s F-12 medium (Sigma, UK), followed by two hours incubation at 37 °C with 5% CO_2_. After incubation, the MTT solution was replaced by 1 ml of 99,8% isopropanol (STANCHEM, Poland). The plates covered with tinfoil have been shaken for 15 min at 100 rpm (ES-20, Biosan), followed by the color change quantification using the plate reader (Synergy H1, BioTek) at 570 nm.

The cell viability was assessed by the formula:

(1) $$Cell\;viability\;\left(\%\right)=\frac{\left({\text{O}\text{D}\;}_{570}\;\text{o}\text{f}\;\text{t}\text{e}\text{s}\text{t}\;\text{n}\text{a}\text{n}\text{o}\text{p}\text{a}\text{r}\text{t}\text{i}\text{c}\text{l}\text{e}\text{s}\right)–\;\left({\text{O}\text{D}}_{570}\;\text{o}\text{f}\;\text{b}\text{l}\text{a}\text{n}\text{k}\right)}{\left({\text{O}\text{D}\;}_{570}\text{o}\text{f}\;\text{c}\text{o}\text{n}\text{t}\text{r}\text{o}\text{l}\right)\;–\;\left({\text{O}\text{D}\;}_{570}\;\text{o}\text{f}\;\text{b}\text{l}\text{a}\text{n}\text{k}\right)}x\;100\%$$

### 2.5 Cells preparation for scanning electron microscopy

The morphology of rat MSC cultivated in the presence of nanoparticles exposed to magnetic field was studied using a Vega Tescan SEM. Before imaging, the cells were fixed in glutaraldehyde, dehydrated with ethanol, dried, and covered with a thin gold (Au) layer in order to avoid charging effects during electron microscopy scanning. The fixation process was done at 4 °C in 2.5 % glutaraldehyde for 12 h, followed by other 24 h in a saline buffered solution (NaCl 0.9 %). The dehydration process involved incubation in gradually increasing ethanol concentrations from 30 % to 97 % at room temperature. Since we were more interested in seeing the cells redistribution under magnetic field influence rather than the cell membrane integrity, the drying process has been performed in normal atmosphere at room temperature. Before imaging, the samples were coated with an ultrathin layer of Au by using a Cressington 108auto sputter coating machine.

## 3 Results and discussion

**Fig. 1:**
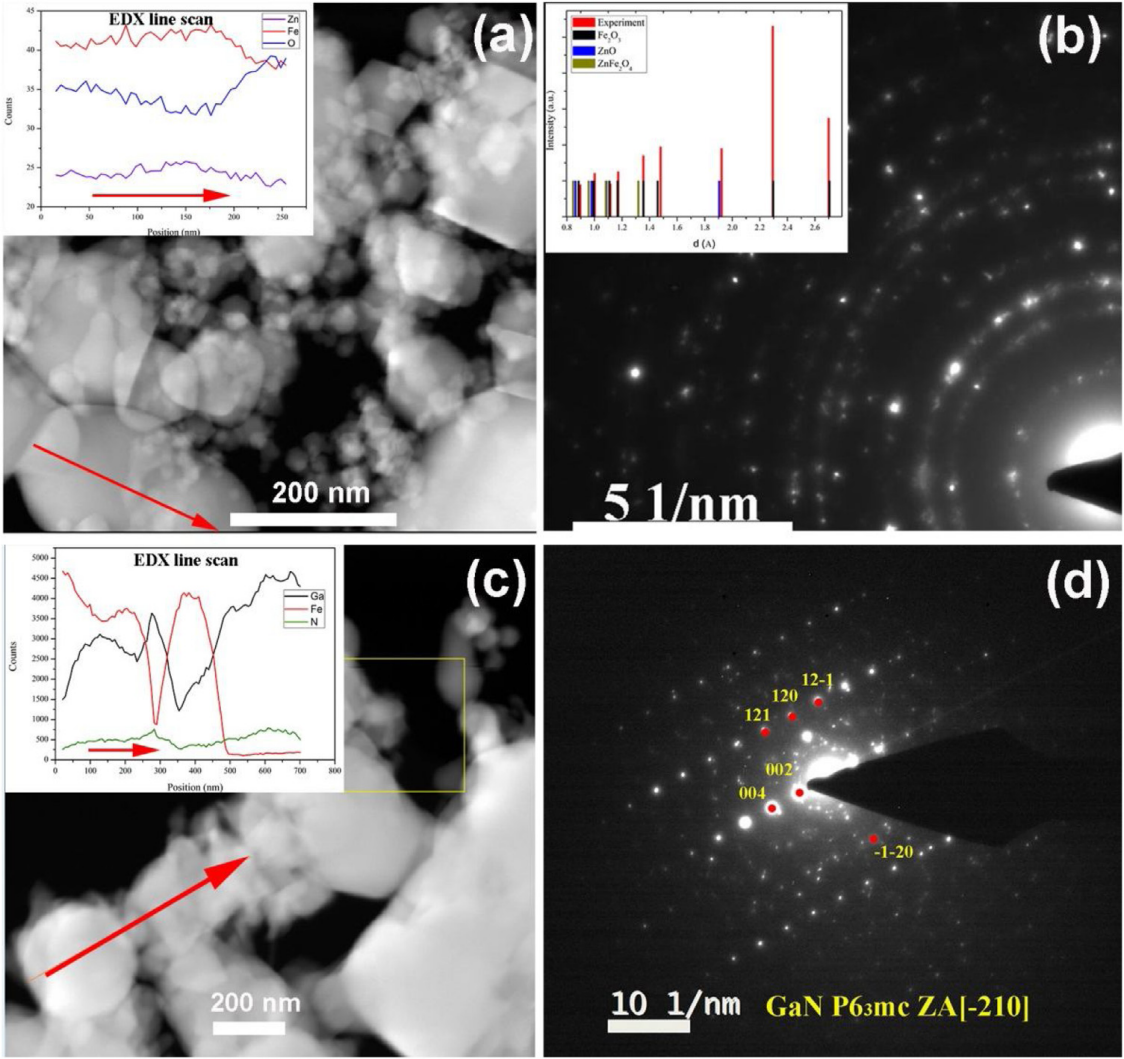
TEM and SAED characterization of initial zinc ferrite nanoparticles (a) and (b), and GaN nanoparticles after the HVPE growth process (c) and (d) respectively. The insets in (a) and (c) represent the EDX scan results of representative nanoparticles. The inset in (b) shows the intensity distribution of d-values of initial nanoparticles, red columns represent experimental results; black - Fe_2_O_3_ (space group R-3c), blue - ZnO (s.g. P63 mc) and dark yellow - ZnFe_2_O_4_ (s.g. Fd-3 m) reference values corresponding to those found in the sample. (For interpretation of the references to colour in this figure legend, the reader is referred to the web version of this article).

The quality of the GaN film grown on the sacrificial ZnFe_2_O_4_ nanoparticles is disclosed by the results of the TEM characterization presented in Fig. 1. From Fig. 1(a) it can be observed that the initial zinc ferrite nanoparticles are separated and vary in sizes from several nanometres to 200 nm. The selected area electron diffraction pattern (SAED) presented in Fig. 1b shows different reflections which appear to be related to particles with different orientations.

The high-quality HVPE-grown GaN layer on zinc ferrite nanoparticles is revealed by TEM measurements presented in Fig. 1(c) and (d). The sacrificial material consists of a mixture of cubic ZnFe_2_O_4_, trigonal Fe_2_O_3_ and wurtzite ZnO nanoparticles, demonstrated by the diffraction pattern analysis (Fig. 1b). In the HVPE growth process, at first stage, the nucleation of GaN starts on the hexagonal ZnO sites, followed by the next growth step at a higher temperature, when the zinc oxide is being reduced due to high temperature and corrosive atmosphere. Energy dispersive X-ray analysis (EDX) line scans (Fig. 1c) confirm the presence of Ga, Fe, N and, in some regions, the presence of oxygen in low quantities. Also, it shows the variation of Fe and Ga intensities along the line of scanning, indicating the formation of GaN shell layer around Fe-based nanoparticles. The high crystallinity of GaN shell is confirmed by the electron diffraction pattern (Fig. 1d), which reveals the wurtzite-type crystal structure.

**Fig. 2:**
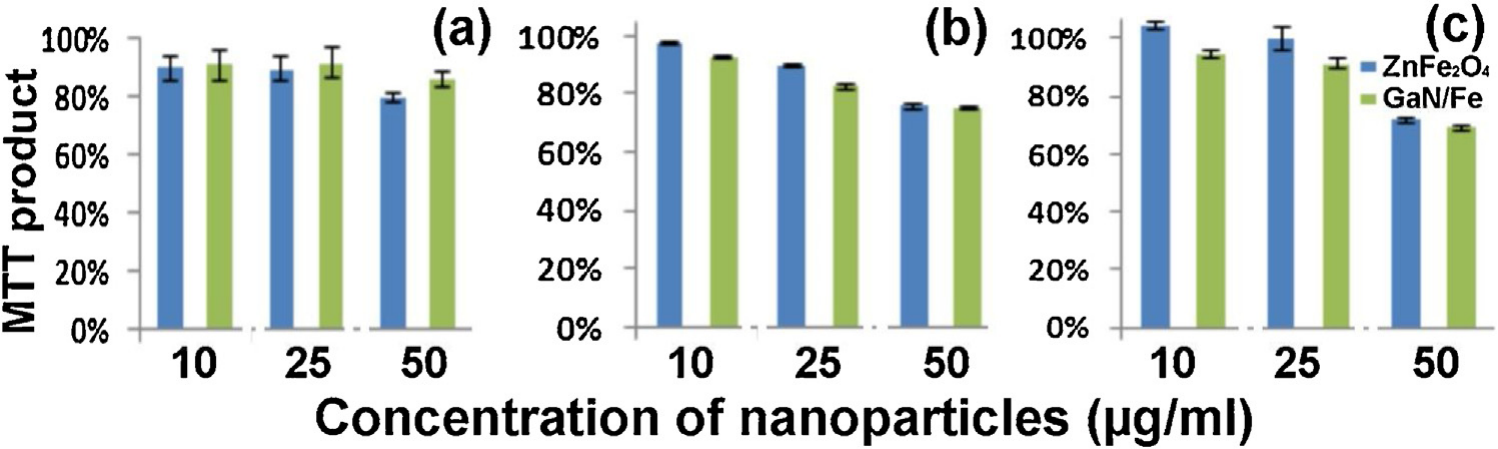
MTT product after (a) 24 h, (b) 48 h and (c) 72 h of incubation of cells with 10, 25 and 50 μg/ml of ZnFe_2_O_4_ and GaN/Fe nanoparticles. The percentage results are reported to the control group, which consist of same amount of cells incubated without any nanoparticles into the culture media.

Fig. 2 depicts the cells dynamic activity revealed by the MTT product collected after 24, 48 and 72 h of incubation of cells with different quantities of nanoparticles.

After one day of incubation of cells with nanoparticles, the cells number and viability were found to be independent upon concentration of nanoparticles in the medium. Incubating cells for three days, a gradual decreasing of cells viability reflected by the MTT product has been observed at a higher concentration of nanoparticles in the medium, independent on particle`s type.

**Fig. 3:**
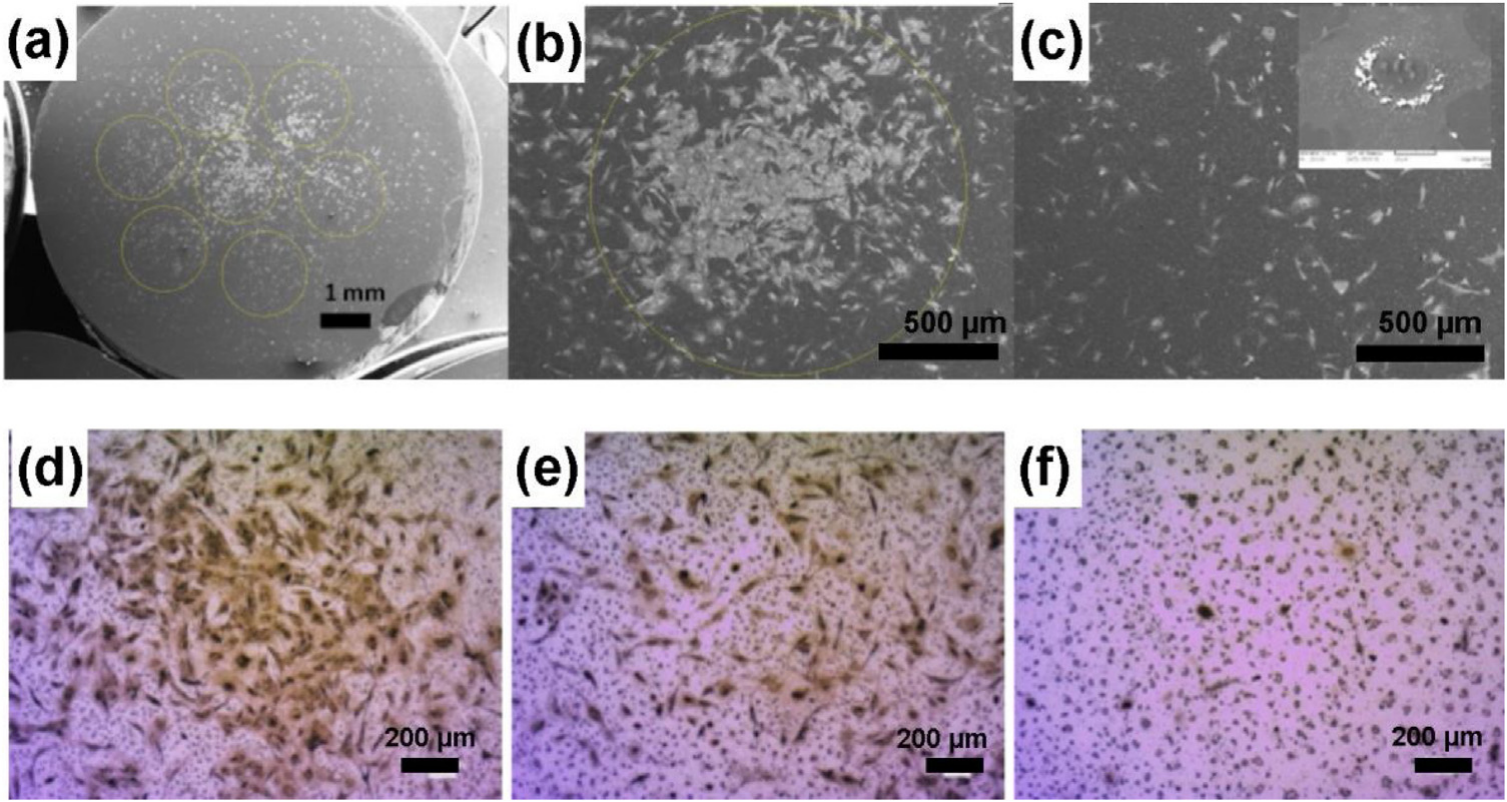
Distribution of MCS loaded with GaN/Fe nanoparticles after 48 h of incubation under magnetic field influence. (a-c) SEM images, (d-f) optical pictures of cells in the region of the magnet, in the near proximity, and several millimeters side away from magnets, respectively. The inset in (c) shows a single cell loaded with nanoparticles.

During the HVPE growth process, the ZnO component from the initial nanoparticles has been replaced by the more stable GaN. A high quantity of Fe_2_O_3_ has also been reduced to Fe, which decreases the overall mass and increases the magnetic properties of resulted nanoparticles. The advantage of GaN/Fe is that low concentration of nanoparticles (10 μg/ml) is enough to carry cells and rearrange them in low intensity magnetic (as low as 200 μT) field, see Fig. 3. The concentration of 10 μg/ml of nanoparticles does not disclose any significant differences in cells number and metabolic activity of cells after three days of incubation (Fig. 2).

**Fig. 4:**
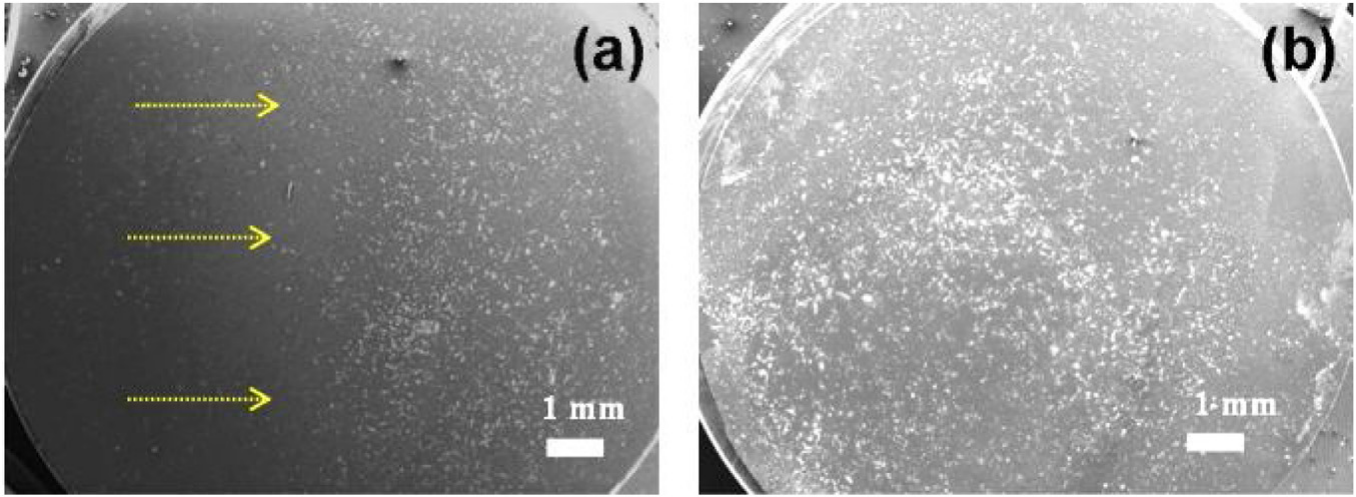
Non-homogeneous distribution of MSC loaded with 25 μg/ml of ZnFe_2_O_4_ nanoparticles under the influence of static magnetic field (a) and in the control group (b).

Fig. 3 shows the tendency of MSC loaded with GaN/Fe nanoparticles to rearrange under the influence of low magnetic field gradient, while the MSC loaded with ZnFe_2_O_4_ nanoparticles could not be influenced in the same experimental conditions. In order to rearrange cells loaded with initial zinc ferrite nanoparticles, a higher concentration of nanoparticles was needed (25 μg/ml), as well as higher magnetic field was applied (1 m T). Fig. 4 depicts the distribution of cells loaded with ZnFe_2_O_4_ nanoparticles and exposed to a magnetic field.

Since the increasing concentration of nanoparticles in the culture media slightly influence the proliferation rate of MSC, it is desirable to keep the concentration of nanoparticles as low as possible. Covering the Fe-based nanoparticles with chemically stable GaN also increases the long-term stability of the material in the intracellular media, which is not the case when ZnO nanoparticles are used.

Along with inherent piezoelectricity of GaN, the magnetic properties related to the high content of Fe make the nanoparticles to become a multifunctional platform for cells imaging, transport and therapy. Tracking cells with magnetic nanoparticles [21] become more attractive since the traditional contrast agents have a relatively short half-life. The remote electrical stimulation of cells by piezoelectric nanoparticles activated with ultrasound field can be used for therapeutic applications, e.g. inhibition of cells proliferation [22] or directed differentiation of cells [23]. By using the GaN/Fe based nanoparticles one can remotely guide cells with nanoparticles and electrically stimulate them.

## 4 Conclusion

In this work, we demonstrate the biocompatibility of semiconductor-based nanoparticles (ZnFe_2_O_4_ and GaN/Fe) with mesenchymal stem cells. It has been shown that low quantities of nanoparticles do not affect the cells metabolic activity while increasing concentrations have an inhibiting influence on the proliferation of MSCs. The magnetic field guiding and redistribution of MSCs loaded with nanoparticles is demonstrated. GaN growth on zinc ferrite nanoparticles increases the chemical stability of the material, which allows the long term monitoring of tracked cells as well as remote electrical influence.

## CRediT authorship contribution statement

**Tudor Braniste:** Conceptualization, Methodology, Writing - original draft, Writing - review & editing. **Vitalie Cobzac:** . **Polina Ababii:** . **Irina Plesco:** . **Simion Raevschi:** . **Alexandru Didencu:** . **Mihail Maniuc:** Conceptualization, Supervision, Writing - review & editing. **Viorel Nacu:** Conceptualization, Supervision, Writing - review & editing. **Ion Ababii:** Conceptualization, Supervision, Writing - review & editing. **Ion Tiginyanu:** Conceptualization, Supervision, Writing - review & editing.

## Declaration of Competing Interest

The authors declared that they have no conflicts of interest to this work. We declare that we do not have any commercial or associative interest that represents a conflict of interest in connection with the work submitted.
